# Time Course of Neural Process of Certain and Uncertain Punishment in Decision Making

**DOI:** 10.3390/bs15111543

**Published:** 2025-11-13

**Authors:** Wenwei Qiu, Huijian Fu, Linanzi Zhang, Qingguo Ma, Lu Cheng

**Affiliations:** 1 College of Economics & Management, China Jiliang University, Hangzhou 310018, China; qww@cjlu.edu.cn; 2 School of Management, Guangdong University of Technology, Guangzhou 510006, China; huijian_fu@gdut.edu.cn; 3 School of Business Administration, Guizhou University of Finance and Economics, Guiyang 550025, China; 11520020@zju.edu.cn; 4 Institute of Neuromanagement Science, Zhejiang University of Technology, Hangzhou 310023, China; 5 Neuromanagement Lab, Zhejiang University, Hangzhou 310058, China; 6 Chinese Academy of Science and Education Evaluation, Hangzhou Dianzi University, Hangzhou 310018, China

**Keywords:** uncertain punishment, event-related potentials, electric shock

## Abstract

Little is known about how people evaluate certain and uncertain punishment. This study utilizes EEG technology to explore the cognitive processing mechanisms involved in the threat of punishment within economic game scenarios. Specifically, it investigates the impact of punishment uncertainty, integrating economic game paradigms with electric shock stimuli. FRN, P300, and SPN reflect the attention and readiness of the neural system during the anticipation of punishment. The results showed that the shock cue elicited a larger FRN and P300 than the uncertain cue, while there was no significant difference in SPN, during the anticipation for potential shock. The self-rating indicated that the uncertain cue triggered the most negative effect, and the pain-related P2 revealed that the uncertain cue increased pain perception, implying that uncertain punishment was more threatening than certain punishment. The single-trial analysis of EEG power with the linear mixed-effects model further supports these findings. This study demonstrates that, by strategically manipulating the uncertainty of the punishment, one can achieve a high-threat effect at a lower cost.

## 1. Introduction

Cooperative behavior is common in primate species (Sussman et al., 2005). Human society is based on large-scale cooperation among genetically unrelated individuals, which sets humans apart from other animals (Henrich, 2003). Cooperation, as a widespread phenomenon in the natural system, ranges from microbes to the whole human societies (Shao et al., 2019). For achieving benefits that cannot be attained by individuals, humans cooperate everywhere (Krasnow et al., 2015). Villagers band together to defend their homeland. Researchers partner with each other for academic collaboration (Krasnow et al., 2015). Cooperation also plays an important part in firms’ innovation process (de Faria et al., 2010), in addition to the essential cooperation and exchanges among countries, whereas problems in cooperation may arise in the meantime, such as low participation, cheating, or free-riding (Krasnow et al., 2015; Raihani et al., 2012; Shao et al., 2019).

With such a crucial role in social stability and as the impetus for promoting social progress, cooperation has become an increasingly concerning issue among researchers (Sachs et al., 2004; Wingreen & Levin, 2006). Several studies have found that some realistic factors and evolving mechanisms such as kin selection punishment and reward have an influence on the cooperative behavior in the well-known “tragedy of the commons” issue (Feeny et al., 1990; Garrett & Ellinckson, 1968). A study also suggested that, by imposing an ex post punishment or reward, the agent will be rendered to perform certain actions congruent with the principals’ intention (Rigdon, 2009). Researchers have given substantial attention to the threat of punishment for its impact on sustaining human cooperation (Kroupa, 2014). Researchers have demonstrated that punishment-based social control was able to solve the conundrum of cooperation (Axelrod, 2009; Fehr & Gächter, 2002; Sigmund, 2007). It has also been proven that punishment is an effective method for promoting cooperation (Helbing et al., 2010; Perc, 2012). The evolutionary puzzle of group cooperation can be solved by the potential power of punishment (Krasnow et al., 2015). In addition, Raihani et al. (2012) found that participants were encouraged to be more cooperative and engage in less cheating by the punishment mechanism. Gurerk (2006) also proposed that punishment mechanisms are more popular with institutions. Two functions were summarized for the threat of punishment. One is the fitness reduction that concerns the direct function of punishment to reduce the relative fitness of free riders. The other puts emphasis on labor recruitment. Free riders are made to cooperate by the mechanism (Krasnow et al., 2015).

As many studies have shown the great impact of punishment on promoting cooperation, some scholars have also paid attention to the problems regarding the mechanism of punishment in promoting cooperation. Rigdon (2009) raised some questions including an efficient way to structure incentives, the real effect of punishment mitigating the agency problem, and what size of “sticks” works best. Moreover, there also exists the puzzle that the cost of punishment may increase with the group size (Krasnow et al., 2015). Krasnow et al. (2015) controlled the cost of punishment as the group size increases by allowing punishment to be probabilistic. The probability of punishment ranges from 0% to 100%, suggesting an uncertain condition where agents do not punish every free rider every time. In this way, the deterrent goal of the threat of punishment can be achieved without the limitation of the group size. Punishment can be divided into two types: certain punishment and uncertain punishment. Certain punishment connotes that the punishment against a specific behavior is predictable, including its occurrence, severity, time course, etc., while uncertain punishment represents a punishment whose features cannot be anticipated beforehand. Although the threat of punishment manages to repel hostile actions, whether certain punishment or uncertain punishment is more effective remains elusive.

In fact, such probabilistic and uncertain punishment inspires us with a novel method that promotes cooperative behavior with respect to the punishment mechanism. Noted to be stressful and dysphoric, uncertainty brings us the feeling of being impotent and having unpredictable control towards our world as well as the things that happen to us (Hogg, 2007). Uncertainty was proven to play a crucial role in the motivation of human behavior and act as an important motive for ideological conviction (Hogg et al., 2010a; Hohman & Hogg, 2015). Studies have proven that the unpredictable threat aroused people’s fear, which, in turn, probably prevented participants from thinking rationally (Fellner & Güth, 2003; van Dijk & Vermunt, 2000). Moreover, according to the uncertainty–identity theory, people tend to be uncomfortable with one’s perceptions, attitudes, values, or feelings under the uncertain context. And uncertainty will motivate humans to reduce self-uncertainty (Hohman & Hogg, 2015). One effective way to reduce self-uncertainty is group identification. Connected with the social categorization which generates in-group identification and regulates perception, feelings, and behaviors for self and others, identification is a depersonalized behavior (Hohman & Hogg, 2015). Hogg et al. (2010b) proposed the following: “Identification reduces uncertainty because it furnishes a sense of who we are, how we should behave, and how others will treat us”. Group identification is an important factor influencing people’s willingness and participation in cooperation. Hence, if we want to strengthen people’s cooperative behavior, strengthening their group identification is needed first. From the above discussion, the implication that, when people are under uncertainty, they will strengthen their group identification was revealed. In this way, this study proposes that, for a person in an uncertain situation, his/her cooperative behavior is also enhanced based on the strength of group identification.

Since the research on probabilistic punishment mainly implies the effectiveness of punishment and the advantage of the cost savings from the perspective of the punisher, the internal mechanisms of this outcome and the impact of uncertain punishment on punished parties remain unclear (Krasnow et al., 2015). Due to the fact that uncertainty can be primed by instant situations (Hogg et al., 2010a), a lab experiment will be employed to investigate the issue.

A revised trust game to induce monetary conflict is designed. In the field of behavioral and experimental economics, a trust game was designed to investigate people’s trust and cooperative behaviors as well as various factors contributing to the trust of investors. Initially proposed by Berg et al. (1995), the trust game is a one-shot game between two anonymous players, an investor and a trustee. The investor is firstly assigned with some tokens and has the option to keep all or invest some of the endowment to the trustee. The tokens invested to the trustee are then multiplied. Subsequently, the privilege goes to the trustee who will make the choice to keep all the multiplied tokens or return a certain amount to the investor. In this way, the trust game can be good to adopt to examine the behavioral and neural features when the trustee is faced with certain kinds of punishment, in the case when the investor is not satisfied with the number of tokens reciprocated and is entitled to exert punishment on the trustee.

This study aims to explore the neural differences between certain and uncertain punishment by using a combination of a behavioral experiment and an event-related potential (ERP) experiment. Participants’ cooperative behavior can be observed through behavioral data, while participants’ cognitive and emotional changes in the decision process can be reflected by neurophysiological indicators. As one of the effective neurophysiological indicators, ERP components could reveal participants’ neural process of certain and uncertain punishment in decision making.

In the ERP study, in addition to the intensity of the stimulus itself, the magnitude of the pain sensation caused by the stimulus is also regulated by the cognitive system. The P2 wave is an effective indicator for objectively evaluating pain perception (Wager et al., 2006). Studies have shown that the P2 wave reflects the perceived intensity of pain rather than the physical intensity of the pain stimulus itself (Naro et al., 2017). The greater P2 is, the more intense the perceived pain is, and there is a correlation between them (Garcí-Larrea et al., 1997). In other words, P2 can be used to objectively evaluate the perceived pain level, and the higher P2 is, the higher the pain level will be. In this study, P2 was employed to measure participants’ pain perception.

However, feedback-related negativity (FRN) reflected the processing of reward prediction error in the cognitive system. The valence of FRN is sensitive to the outcomes, which means the benefit and loss will induce a different FRN. Specifically, in the case of a loss, the FRN wave is greater than in the case of a benefit (Cohen et al., 2007). Therefore, the FRN wave can be employed to evaluate the difference of the valence between the different results.

Stimulus-preceding negativity (SPN) is a slowly changing brain wave that is immune to the motor response. It is the EEG change before the event stimulation and reflects the expected process of the cognitive processing system towards stimulation (Brunia & Damen, 1988). SPN is often used by researchers to investigate the role of the information that was used to predict future aversive stimuli in the anticipation stage (Böcker et al., 2001; Brown et al., 2008).

Therefore, the perceived pain of uncertain punishment and certain punishment on the punished ones can be obtained through the change in P2 amplitude. And the hypothesis can be posited as follows:

**H1.**  *The P2 wave induced by uncertain punishment will be significantly greater than that induced by certain punishment.*

In addition, in order to compare the effect of the differences of two kinds of punishment and their brain mechanisms from the perspective of the punished ones, a comprehensive evaluation system was established. The psychological and physiological indices include the level of stress and pain sensation of the punished ones, whereas the behavioral index was the proportion of the improvement in the return amount for investors in each trial. Concluding from previous studies, this study may speculate that, from the perspective of the punished one, the threat of uncertain punishment is greater than certain punishment. In this way, combining with the evaluation index proposed before, the following hypotheses are posited:

**H2.**  *Uncertain punishment arouses a higher level of subjective stress than certain punishment.*

**H3.**  *Uncertain punishment elicits more negative SPN than certain punishment.*

The P300 wave is an ERP component associated with decision-making processes. An enhanced P300 amplitude exhibits a positive correlation with the intensity of an individual’s confidence during decision-making (Pirtošek et al., 2009). Furthermore, the Motivational Significance Theory of P300 proposes that the P300 amplitude is not merely governed by the stimulus probability or task relevance, but rather by the motivational appraisal value of a stimulus, namely, its capacity to upregulate or downregulate behavior in relation to current goals (Nieuwenhuis et al., 2005). In the current investigation, both no punishment and certain punishment, despite possessing opposing valences, represent unambiguous feedback with a high informational value. They furnish clear signals for decision-making, robustly prompting the maintenance or adjustment of ongoing behavioral strategies. This certainty confers heightened salience to these stimuli within the task context, thereby mobilizing increased attentional resources for processing, which may ultimately be reflected in larger P300 amplitudes (Johnson, 1986). In contrast, the ambiguity introduced by the uncertain punishment condition precludes the provision of an efficient behavioral guide. Based on the foregoing rationale, the current study proposes the following hypothesis:

**H4.**  *Certain punishment elicits a larger P300 than uncertain punishment.*

**H5.**  *The proportion of the improvement in the return amount after uncertain punishment will be higher than after certain punishment.*

A recent study has demonstrated that, when there is a mere imposition of the material costs as punishment, the punishment is not always effective for compliance promotion (Villatoro et al., 2014), while physiological pain sensation is one of the most basic physiological phenomena of human beings, excepting very few people who cannot feel pain due to physiological abnormalities, the vast majority of people are able to suffer the effects of pain. Pain has an evolutionary significance because it is often a sign of injury and is associated with an existential threat. Pain stimuli are quite negative for the organism, and it is something that the organism tries to avoid, in terms of evolution. Therefore, exerting pain stimuli is a sensitive punishment method for most people. The sense of pain directly affects the sensory organs of the participant (such as the skin on the arm), and can elicit withdrawal reactions without cerebral cortex processing. Therefore, as a direct form of punishment, pain stimuli were employed as the punishment method for participants in this experiment.

To further substantiate the influence of uncertain punishment on dynamic cognitive processing and to elucidate the complex dynamics of electroencephalographic (EEG) signals (Grandchamp & Delorme, 2011; Ma et al., 2016), the present investigation utilized a time–frequency analysis to examine the cerebral oscillatory activity and uncover the underlying neural mechanisms of cognitive operations. This analytical approach facilitates the exploration of reactivity to affective stimuli through the assessment of synchronous neural oscillations, enabling the detection of subtle differentiations in emotional processing (Aftanas et al., 1996). The methodology has been extensively applied in probing neurophysiological responses to emotionally salient stimuli (Balconi & Lucchiari, 2007). Notably, oscillatory activities within the alpha (8–13 Hz) and beta (13–30 Hz) frequency ranges have been broadly implicated in emotional processing (Schubring & Schupp, 2019; Tsang et al., 2001). Substantial research indicates that power within these frequency bands increases during the presentation of emotionally arousing stimuli (Güntekin & Basar, 2007; Mennella et al., 2017; Uusberg et al., 2013). For instance, the exposure to emotional imagery elicits enhanced beta oscillatory activity across the frontal, central, and parietal regions (Güntekin & Başar, 2010). Comparative analyses reveal that negative images, relative to neutral counterparts, induce significantly augmented beta-band power (Woodruff et al., 2011). Furthermore, high-arousal affective picture stimuli evoke heightened EEG synchronization in the beta band between the posterior parietal and prefrontal electrode sites (Miskovic & Schmidt, 2010). Building upon this evidence, the current study employed measures of alpha and beta power to investigate the neural oscillations associated with the effects of both certain and uncertain punishment on an individual’s emotional anxiety.

Additionally, an analysis of the theta rhythm was incorporated, capitalizing on its sensitivity to mnemonic influences to investigate the potential inter-trial dependencies in subjective ratings of anxiety. The theta rhythm constitutes a neural oscillation that has been robustly linked to cognitive processes including learning and memory in mammalian species (Seager et al., 2002; Winson, 1978). Empirical evidence consistently indicates a direct relationship between frontal theta wave activity and cognitive task demands; specifically, the spectral power of the theta frequency increases proportionally with task difficulty during periods of focused attention, information processing, and memory encoding or retrieval (Craig et al., 2012). Furthermore, elevated theta activity has also been associated with heightened states of mental fatigue. During specific short-term memory tasks, a pronounced activation within the theta frequency band is observable in the hippocampal formation (Vertes, 2005).

## 2. Materials and Methods

### 2.1. Participants

Sample size was determined with G*Power 3.1.9 for a 3 × 4 fully within-subjects ANOVA. Assuming α = 0.05, power = 0.95, a medium effect size (Cohen’s f = 0.25), average correlation among repeated measures ρ = 0.50, and ε = 1 (sphericity met), the required sample size was 18. Allowing for a 20% attrition rate, 23 participants were recruited.

Twenty-three students were recruited via advertisement posted on the online bulletin board system of Zhejiang University to participate in this study. Data of two participants were rejected since they gave back CNY 0 in every trial. As a result, data of 21 subjects (8 females, with a mean age of 21.76 ± 1.7) were included in data analysis. All participants were right-handed, and had normal or corrected-to-normal vision. Written informed consent was obtained before the experiment, and they were paid for participation after the experiment. The study was approved by the Ethics Committee of Neuromanagement Laboratory of Zhejiang University.

### 2.2. Experimental Procedure

**Figure 1:**
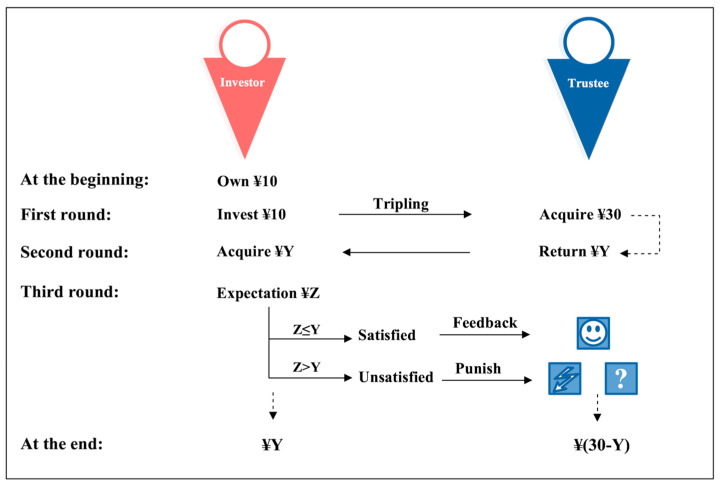
Economic game task diagram.

For the purpose of capturing the subjective evaluation of the cues for punishment, this study mainly focused on the role of trustee. The major difference between the task in current study and classical trust game lies in the punishment stage, which is implemented after trustee decides how much money is to be paid back to the investor (see Figure 1).

In order to avoid introducing interfering variables, the amount invested by the “investor” to the “trustee” in each trial is fixed at CNY 10.

**Figure 2:**
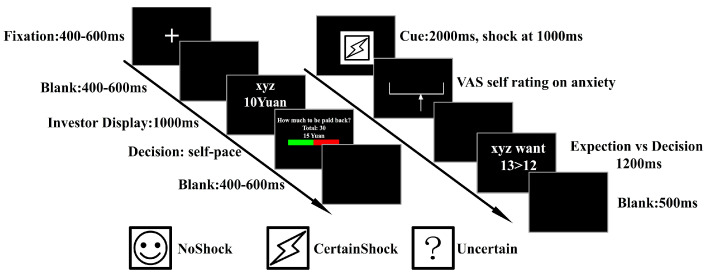
Experimental schema.

After the decision made by the trustee, a cue appeared at the center of the computer screen, indicating investor’s reaction to player’s payback. There were three kinds of cues: NoShock, Shock, and Uncertain (see Figure 2). The NoShock cue signaled that the money returned by trustee was equal to or higher than investor’s expectation, such that no hostile action against trustee would be implemented. The Shock cue meant that the payback could not meet investor’s expectation, so an electric shock would be exerted, while, when an Uncertain cue appeared, either an electric shock or non-shock would be implemented. The intensity of the electric shock remained constant throughout the experiment, and was determined according to a pretest on each participant (the trustee), ensuring that it could successfully elicit participant’s intense sense and did not exceed his/her tolerance.

Participants were instructed to act as trustees. Each trial began with a fixation in the center of the screen. After receiving an investor’s CNY 10 and turning it into CNY 30, the trustee had to decide the amount of money to be paid back to the investor. Then, one of three cues would appear, indicating investor’s intention to punish the trustee or not. A smile icon serves as NoShock cue, a lightning icon as Shock cue, and a question icon as cue for Uncertain. The icon images are drawn with black lines on a white background. Three images are with similar luminance (241.8, 243.1, and 245.7 gray level) and contrast (54.4, 51.8, and 45.9 gray level). Following the implementation of shock or non-shock, the trustee was asked to rate the level of anxiety at the moment of seeing the cue, via a visual analogue scale (VAS). Finally, a contrast of the expected monetary payback of the investor against the actual payback was presented.

Participants were required to complete this revised one-shot trust game while an electroencephalogram (EEG) was recorded. All participants had to complete 3 blocks of a total of 90 trials in the game, and, in each trial, they met a new investor, whose expectation of monetary payback was manipulated by the computer program to ensure that each cue appeared approximately 30 times.

Due to the low signal-to-noise ratio characteristic of ERP technology, it is necessary to average multiple trials to eliminate random EEG noise and extract useful EEG signals. Therefore, the experimental design must ensure that each experimental condition has a sufficient number of valid trials (at least 20 trials) to meet the requirements for ERP analysis and obtain scientifically valid conclusions. This study adopted the following method to ensure and balance the number of trials across experimental conditions:

In each trial, the number of occurrences of three conditions (no shock, certain shock, and uncertain shock) is sorted in ascending order. If the difference between the maximum and minimum values is less than or equal to 3, one of the three conditions is randomly presented. If the difference is greater than 3, the condition with the fewest occurrences is presented. When participant’s return value is at an extreme (CNY 30 or 0), the above rules do not apply. Specifically, when participant returns the full amount (CNY 30), the current trial’s result will inevitably be no punishment, indicating that the investor is satisfied with the outcome and will not punish. When participant returns 0, any of the three conditions may occur, but the probability of no punishment is only 9.09% (1 in 11), indicating that the investor is highly likely to take punitive action (certain shock or uncertain shock) towards this return value. The probability of exerting electric shock after uncertain shock cue is 50%.

In each trial, the program receives the return value confirmed by participant’s button press and determines the experimental condition to be presented according to the rules mentioned above. Based on the return value and the experimental condition, the possible range of trustor’s expected value can then be determined. For example, if participant’s return value is CNY 15 and the experimental condition is no shock (satisfied), the range of the investor’s expected value is 0 to CNY 15. If participant’s return value is CNY 15 and the experimental condition is certain shock or uncertain shock (not satisfied, punishment applied), the range of trustor’s expected value is CNY 15 to 30.

Then, using a normal distribution with a mean of CNY 15 and a standard deviation of CNY 5, each integer within the range of the expected value is assigned a corresponding weight according to the probability distribution, and a random selection is made to determine trustor’s expected value for the current trial. Specifically, the investor’s initial expected value follows a normal distribution (mean = 15, standard deviation = 5). Once the range of investor’s expected value is determined, an integer is drawn from the integers within that range, according to their probabilities in the original normal distribution. The drawn integer serves as investor’s expected value of current trial. By using this method, the expected values in each trial will be more normal and reasonable.

During the experiment, participants sat comfortably in a chair in front of and 1 m away from a 17-inch CRT screen and in an electrically shielded and acoustically isolated room. Participants made decisions by pressing buttons of a keypad with right hand. An electrical stimulation device (mode: YRKJ-F1002; Yiruikeji Co. Ltd., Zhuhai, China) was used to exert electrical shock, by means of an electrode attached to the dorsum of the participant’s left forearm. To minimize participants’ fatigue and habituation, the electrode attached on the forearm was moved to another area nearby for following block, while the participant took a 10 min rest between blocks. The duration of electrical shock was 1 ms, and the level of electrical intensity was set according to the individual endurance, which was measured by an endurance test before the experiment. In the test, participants received increasing electrical shock starting from 1 mA with increments of 1 mA and with time interval of about 10 s, until participants felt unbearable. The 75% of unbearable intensity was used as the intensity of punishment shock in the experiment (mean = 25.77 ± 11.97 mA).

### 2.3. Electroencephalographic Recordings and Data Analysis

Electroencephalographic (EEG) was recorded with an electrode cap with 64 Ag/AgCl electrodes mounted according to the extended international 10/20 system, with an online bandpass filter from 0.05 to 70 Hz and the 50 Hz notch filter to avoid power line contamination, and sampled at 1000 Hz using Neuroscan Synamp2 Amplifier (Scan 4.3.1, Neurosoft Labs, Inc., Sterling, VA, USA). Eye blinks were recorded from left supra-orbital and infra-orbital electrodes, while the horizontal eye movement EEG was recorded from electrodes placed 1.5 cm lateral to the left and right external canthi. The ground electrode was placed at forehead (at the location of electrode AFZ). The reference electrode was attached to the left mastoid. Every electrode impedance was maintained below 5 kΩ.

The raw EEG data was firstly inspected visually for artifacts, and then it was re-referenced offline to linked mastoid electrodes by subtracting from each sample of data recorded at each channel one-half the activity recorded at the right mastoid.

EEG segmented from 400 ms before the onset of cues to 1000 ms after the time point for potential electrical shock was extracted for further analysis. In other words, the time window of epoch was −400 ms to 2000 ms, with the onset of cues as 0 ms. Electrodes with poor data were removed. An independent component analysis (ICA) was implemented on the remaining data, and then the eye movement components and artifact components were removed. After the removal of ocular and artifact component, the EEG data of the deleted electrodes were interpolated by EEGLAB function. Then, data were filtered with 30 Hz low-pass filter. A baseline correction was applied on the epochs, with data from −400 ms to 0 ms as the baseline. All trials in which EEG voltages exceeded a threshold of ±100 μV during the recording epoch were excluded from analysis. Data for each subject met the criterion that at least 20 out of 30 trials per condition were retained after the rejection process (average number of valid trials: NoShock = 28.48 ± 2.64, Shock = 26.86 ± 3.97, and Uncertain = 27.95 ± 2.44).

**Figure 3:**
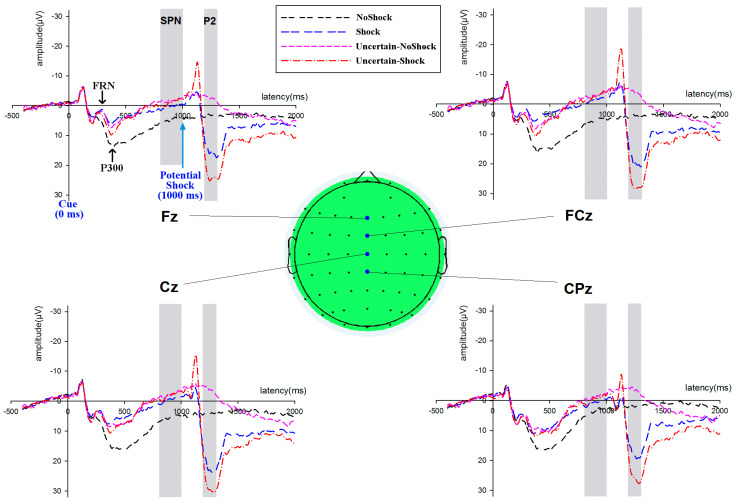
The grand average ERP waves at midline electrodes.

The maximum amplitude within 300 ms to 600 ms window was measured as the value of P300. Since FRN was overlapped by P300, in order to reduce such influence, the difference wave method was employed on FRN measurement. The difference wave between each cue was calculated on each participant, and the mean amplitude from 200 ms to 350 ms was measured as FRN amplitude. Brain wave data from the window of 800 ms to 1000 ms within the epoch, preceding the potential electrical shock time point, was measured as SPN (see Figure 3). For each condition of each subject, the peak latencies of pain-evoked P2 were identified at the electrode (CZ) where the P2 peak showed the maximum amplitude. Average amplitude within a 20 ms window centered on this latency was measured as the P2 amplitude (Brown & Jones, 2010; Kakigi et al., 2000).

For EEG power analysis, this study performs a Morlet wavelet transform on raw EEG amplitudes, with the following parameters: frequency range from 4 Hz to 30 Hz, bandwidth of 0.5 Hz, and the mother wavelet set to have a 2 s resolution (half-width) at 1 Hz. Through wavelet transformation, power values of each frequency band are calculated for each time point, at each electrode, of each trial. The power values of theta (4–8 Hz), alpha (8–13 Hz), and beta (13–30 Hz) are used as indicators of brain activities for subsequent analyses.

The grand average across participants of ERP for each condition at four electrodes is shown above. The onset of Cue is set at 0 ms, and potential shock happens at 1000 ms. Time windows for SPN and P2 components are signified by gray color in the image. NoShock indicates there is no punishment, and Certain-Shock indicates definitive shock punishment. Uncertain-NoShock represents there is no punishment after the uncertain cue, and Uncertain-Shock represents there is electric punishment after the uncertain cue.

## 3. Data Analysis and Results

The ANOVA analysis was implemented with SPSS Statistics v23, and Greenhouse–Geisser correction is applied if the assumption of homogeneity of variance is violated. The Bonferroni correction was used for multiple comparisons.

### 3.1. Behavioral Data

#### 3.1.1. The Effect of Electric Shock on the Change in Return Amount

At each trial, participants received a cue and a possible electric shock (punishment) before making their next decision. Although participants were informed beforehand that each trial was independent, their decisions could still be influenced by the punishment received in the previous trial. To examine the impact of the previous trial’s punishment on the decision in the subsequent trial (i.e., whether the punishment in the previous trial altered the return amount in the next trial), this study extracted participants’ return amount from the second to the thirtieth trials at each block (a total of 87 trials per participant, 29 trials per block × 3 block).

The change in the return amount was calculated by subtracting the return value of the previous trial from the current trial’s return amount:

Changes in return amount = Current trial return amount − Previous trial return amount

The certainty and shock status of the previous trial were categorized (NoShock, Certain-Shock, Uncertain-NoShock, and Uncertain-Shock). The proportion of trials in which the change in the return amount was greater than 0 (i.e., the return amount increased) was calculated for each category.

Proportion of increased return amount = (Number of trials with change in return amount > 0)/ Total number of trials in that category

A repeated-measures ANOVA was conducted with the proportion of the increased return amount as the dependent variable, and the certainty and shock status of the previous trial as the independent variables (two levels of certainty: certain and uncertain; two levels of shock: shock and no shock). The results indicated that the main effect of certainty was not significant, *F*(1, 20) = 3.542, *p* = 0.074, η^2^ = 0.15; the main effect of shock was significant, *F*(1, 20) = 8.816, *p* = 0.008, η^2^ = 0.306, with a higher proportion of the increased return amount following an actual shock; and the interaction between the two factors was not significant, *F*(1, 20) = 2.194, *p* = 0.154, η^2^ = 0.099.

#### 3.1.2. Results of Self-Ratings of Anxiety

Self-rating results (see Table 1) show how anxious the players were when encountering different cues.

The repeated ANOVA on subjective anxiety data revealed that the main effect of the cue type is significant, *F*(1.54, 30.76) = 44.91, *p* < 0.001, η^2^ = 0.692 (see Table 2). Pairwise comparisons showed that Uncertain-NoShock and Uncertain-Shock elicited higher anxiety than Shock, which, in turn, elicited higher anxiety than NoShock. And there was no significant difference between Uncertain-NoShock and Uncertain-Shock, which suggests that, though players performed the ratings after the occurrence of a shock, they were capable of rating the feeling towards the uncertain cue while getting rid of the influence of whether the shock actually occurred or not.

In conclusion, the uncertain shock cue elicited the strongest perceived anxiety, while the NoShock cue elicited the least.

### 3.2. ERP Data

**Figure 4:**
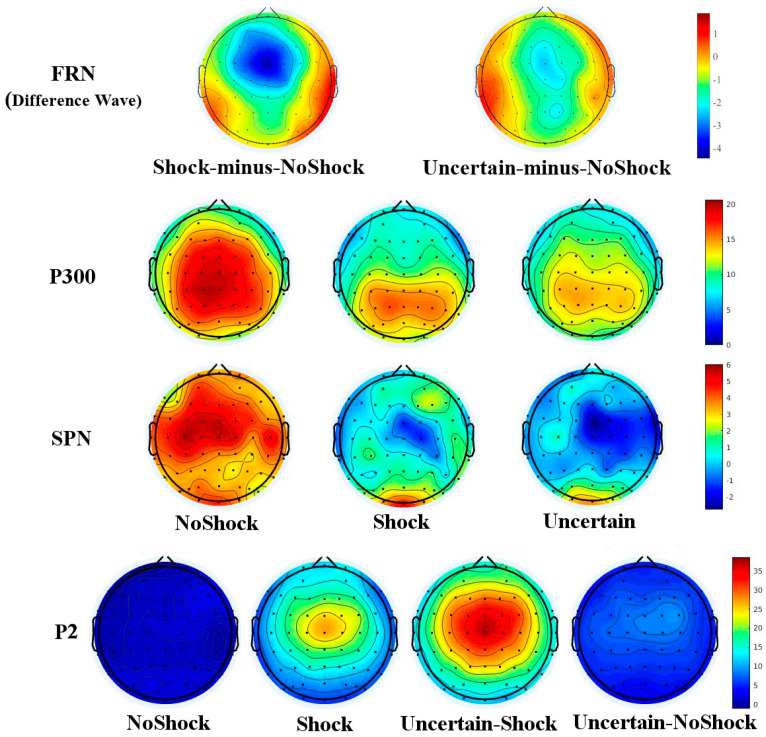
Scalp map of ERP components.

The scalp map shows that P300 dominated mostly in the parieto-occipital region for the Shock and Uncertain conditions (see Figure 4). Data of the region (P3, PZ, and P4) was included in the analysis on P300. A repeated ANOVA with a 3 (Cue: NoShock, Shock, and Uncertain) × 3 (Electrode: P3, PZ, and P4) design was implemented. It revealed that the main effect of Cue was significant [*F*(1.34, 26.8) = 11.81, *p* = 0.001, η^2^ = 0.371]. Pairwise comparisons indicated that there was no significant difference between NoShock and Shock, and both were higher than the Uncertain condition. The main effect of the Electrode was not significant [*F*(1.55, 30.94) = 0.84, *p* = 0.414, η^2^ = 0.04]. The interaction effect was not significant [*F*(4, 80) = 2.22, *p* = 0.074, η^2^ = 0.1].

The difference waves of Shock minus NoShock, and Uncertain minus NoShock were obtained. A 2 (Cue: Shock-minus-NoShock and Uncertain-minus-NoShock) × 4 (Electrodes: FZ, FCZ, CZ, and CPZ) repeated-measures ANOVA on FRN amplitudes revealed that the main effect of the Cue was not significant [*F*(1, 20) = 3.23, *p* = 0.087, η^2^ = 0.139]. The main effect of the Electrode was significant [*F*(1.82, 36.48) = 7.93, *p* = 0.002, η^2^ = 0.284]. The interaction effect was significant [*F*(3, 60) = 3.90, *p* = 0.013, η^2^ = 0.163]. Further examination of the interaction showed that the Shock cue elicited a lower FRN amplitude (larger FRN) than the Uncertain cue at FZ (*t*(20) = −2.22, *p* = 0.038), and FCZ (*t*(20) = −2.47, *p* = 0.023), but not at CZ (*t*(20) = −1.35, *p* = 0.193), and CPZ (*t*(20) = −0.47, *p* = 0.642).

A 3 (Cue: NoShock, Shock, and Uncertain) × 4 (Electrodes: FZ, FCZ, CZ, and CPZ) repeated-measures ANOVA on SPN revealed that the main effect of the Cue was significant [*F*(2, 40) = 13.6, *p* < 0.001, η^2^ = 0.405]. Pairwise comparisons indicated that the difference between Uncertain and Shock was not significant: both of them elicited more negative SPN than NoShock. The main effect of the Electrode was not significant [*F*(2.4, 47.94) = 1.37, *p* = 0.265, η^2^ = 0.064]. The interaction effect was significant [*F*(3.63, 72.64) = 3.61, *p* = 0.012, η^2^ = 0.153].

Since the pain-evoked P2 was elicited by an actual electrical shock, only the data of Shock and Uncertain-Shock were included in the analysis of P2. A 2 (Cue: Shock and Uncertain-Shock) × 4 (Electrodes: FZ, FCZ, CZ, and CPZ) repeated-measures ANOVA on P2 revealed that the main effect of the Cue was significant [*F*(1, 20) = 59.69, *p* < 0.001, η^2^ = 0.749]. The Uncertain-Shock elicited a larger P2 than Shock. The main effect of the Electrode was significant [*F*(1.93, 38.6) = 15.96, *p* < 0.001, η^2^ = 0.444]. Pairwise comparisons indicated that (FCZ = CZ) > (FZ = CPZ). The interaction effect was not significant [*F*(2.07, 41.33) = 0.82, *p* = 0.453, η^2^ = 0.039].

From the brain topography, it can be observed that the strongest activation points of both FRN and SPN components are located in the four midline fronto-central electrodes (FZ, FCZ, CZ, and CPZ) region. From the P300 topography, under the NoShock condition, a large central parietal region is active; in the Shock and Uncertain conditions, the activated areas are mainly located in the parieto-occipital region. The topography also shows that the most active sites for the P2 wave induced by both Shock and Uncertain-Shock are located at the top of the head (FCZ and CZ electrodes), with the P2 amplitude gradually weakening from the central top area towards the periphery.

### 3.3. Mixed-Effects Model

The results of the ERP analysis reveal that EEG activity dominates at PZ within 300–600 ms, at FCZ within 800–1000 ms, and at CZ within 1100–1300 ms.

Based on these results, we calculated the average power of theta (4–8 Hz), alpha (8–13 Hz), and beta (13–30 Hz) within three time windows at corresponding to the most active electrode (300–600 ms at PZ, 800–1000 ms at FCZ, and 1100–1300 ms at CZ) for each trial of each participant. Then, nine power indices (3 bands × 3 windows) were obtained.

Then, based on the single-trial data, we build a mixed-effects model with the following formula:

Anxiety ~ Amount + isShocked + isCertainShock + isUncertain + 9_power_indexes + (1|Participant)

where Anxiety is the subjective rating on anxiety, and Amount is the payback from the participant. isShocked, isCertainShock, and isUncertain are dummy variables indicating whether an actual electrical shock is exerted, whether the cue is CertainShock, and whether the cue is Uncertain, respectively. The 9_power_indexes variable represents the nine power indices described before. (1|Participant) means the participant is defined as a random effect.

The results reveal that the coefficients of the following variables are significant: Amount, isCertainShock, isUncertain, Beta within 300–600 ms, and Alpha within 1100–1300 ms (see Table 3).

## 4. Discussion

In this study, the direct effect of participants’ threat of punishment were manifested in intense negative emotions and the sensation of pain induced by electric shock punishment. These were, respectively, assessed through subjective anxiety ratings and the amplitude of the P2 wave elicited by pain. During the interval between the appearance of the threat cue and the execution of the punishment, the primary effect of the threat was to induce negative emotions, which participants reported retrospectively through a self-assessment. Conducting a retrospective self-assessment after the occurrence of punishment poses the major challenge of potentially being influenced by the actual administration of the shock. However, the subjective anxiety data indicate that participants were able to evaluate their anxiety levels during the anticipated punishment phase, independent of the actual shock occurrence, suggesting that participants in this study could accurately report their emotions. Additionally, the analysis of the anxiety data concludes that, during the anticipation phase, uncertain punishment evoked stronger anxiety in participants compared to certain punishment.

When it comes to actual punishment, the direct effect of the threat-based punishment is reflected in the extent of the harm experienced by the punished individual. In this experiment, the level of harm was measured by the intensity of pain felt by participants during the cognitive processing of the punishment, with the amplitude of P2 serving as the measurement indicator. The analysis of the P2 data showed that uncertain punishment resulted in higher pain levels from the shocks compared to certain punishment. Thus, the uncertainty of the punishment increased the participants’ perception of pain.

The current study aimed to explore the neural underpinnings of processing certain and uncertain punishment. FRN is sensitive to the valence of feedback: typically, negative outcomes elicit a more negative FRN than positive outcomes (Cohen et al., 2007). Therefore, it can be drawn that, at the early phase, the cue for certain shock was perceived as signaling the most negative outcome.

The present study revealed that the stimulus-preceding negativity (SPN) amplitudes elicited in the uncertain punishment and certain punishment conditions did not differ significantly, and both were significantly larger than that in the “no punishment” condition. This finding does not support our hypothesis H3. A potential explanation may lie in the fact that the SPN primarily reflects anticipatory attention and emotional preparation for events of high motivational significance, rather than representing a specific response to stimulus valence (whether positive or negative) (Brunia et al., 2011). Specifically, certain punishment, as a highly arousing negative outcome, carries an explicit threat that effectively mobilizes the individual’s alerting system, thereby capturing substantial cognitive and affective resources during the anticipatory stage (van Boxtel & Böcker, 2004). Similarly, the risk and suspense inherent in the uncertain punishment condition also constitute a potent source of arousal. Uncertainty itself can heighten an individual’s anxiety levels and cognitive load, as the brain must prepare for multiple potential outcomes and sustain a state of readiness. This high degree of motivational engagement is likewise manifested as an enhanced SPN amplitude (Zheng et al., 2015; Deng et al., 2023). Therefore, although the uncertain and certain punishment conditions differ in nature, the intensity of motivational arousal they elicit during the anticipation phase is likely comparable, which accounts for the absence of a significant difference in SPN amplitudes between them. In contrast, the “no punishment” condition, being a neutral event with low motivational salience, fails to elicit a comparable degree of anticipatory resource allocation, thus resulting in the smallest SPN.

To gain a deeper understanding of participants’ subjective feelings of anxiety when faced with certain and uncertain punishment, and to verify the validity of the self-reported data in this study, we adopted a multi-modal data analysis approach. In addition to behavioral self-ratings, we performed a single-trial spectral analysis of the EEG data and constructed a linear mixed-effects model. This model used trial-by-trial self-reported anxiety ratings as the dependent variable, while neural oscillatory activity (alpha and beta band power) reflecting different cognitive and emotional processes was included as key predictors. This approach allowed us to directly examine the relationship between subjective reports and immediate brain activity at the level of individual trials.

The mixed-effects model revealed a key finding: both alpha and beta neural oscillation power showed significant independent positive effects on the self-reported anxiety ratings. Extensive literature indicates that increased beta band (13–30 Hz) activity is closely associated with heightened arousal, anxious states, and intensified negative emotional processing. Meanwhile, alpha band (8–13 Hz) activity over the parietal-occipital regions has traditionally been linked to relaxed states; however, its reduction or context-specific enhancement in situations such as anticipatory anxiety or threat processing has also been interpreted as a marker of emotional and attentional engagement. Our data, showing a positive predictive relationship of both alpha and beta power with anxiety ratings, strongly suggest that, during the anticipation period and after punishment implementation, the neural activity related to emotional arousal and anxiety was significantly modulated. This provides objective physiological evidence that the subjective emotional experiences reported by participants during the task are supported by underlying neural activity. Of particular importance is the precise temporal alignment between the neural oscillations used in our spectral analysis and specific time-domain ERP components. Specifically, the time window of the beta wave activity corresponded to the P300 component, while the time window of the alpha wave activity was linked to the pain-related P2 component.

In summary, the mixed-effects model provides strong evidence that participants’ anxiety ratings were not merely rough, after-the-fact estimates, but were closely tied to genuine psychophysiological states of anxiety experienced during the anticipation and perception of punishment. This finding substantially strengthens the robustness of our conclusions and supports the validity of self-reports as a measure of the internal emotional experience under the present paradigm.

Based on the theory of expected utility (von Neumann & Morgenstern, 1944), the uncertain cue for a potential electric shock could be understood as a cue with negative value, but it was less negative than a certain cue for electric shock. However, such a hypothesis was not consistent with the SPN and self-rating results in the current study, which implied that the uncertain cue triggered the most intense negative emotion.

Uncertainty aversion, or the pursuit of certainty, is one of the crucial characteristics of human beings, since certain situations provide more insurance of security and stability. The current study confirmed both behaviorally and neurally that the threat of uncertain punishment elicited a more negative effect than certain punishment, which implied a more effective strategy of using the threat of uncertain punishment to handle conflicts. Compared with a previous study that allowed punishment to be probabilistic and thus lowered the cost of punishment when the group size increased (Krasnow et al., 2015), this study mainly compares the difference between uncertain punishment and certain punishment from the micro-perspective, discussing the influence of uncertain punishment on human physiology, psychology, and cooperative behavior, as well as comparing the difference in neural mechanism between the two conditions.

Through a neural indicator, for which the main component is P2, this study found that people felt a greater pain sensation when they were undergoing uncertain shock than when undergoing certain shock, even though the electrical shocks were of the same magnitude. The result suggests that the participant’s inner fear and anxiety result in intensifying the pain sensation (Pan et al., 2019). And this was proven by the difference in their subjective anxiety levels under two punishment condition. Uncertainty, indeed, has a significant effect on one’s mental state, which is consistent with the notion raised by Lovecraft: “The oldest and strongest emotion of mankind is fear, and the oldest and strongest kind of fear is fear of the unknown.” The threat of uncertain punishment is stronger than certain punishment.

As for the effect of the two punishments, the comparison of the anxiety level and pain sensation both manifest that the negative effect of uncertain punishment is stronger than that of certain punishment. These results all prove that uncertain punishment can not only reduce the cost of punishment, but also improve the threat effect of punishment.

In terms of the effect of promoting cooperative behavior, a behavioral data analysis showed that both punishments significantly improved participants’ cooperative behavior compared with the condition of no punishment. However, when we compare the proportion improvement in participants’ return amount after the two kinds of punishments, there was no significant difference between the uncertain punishment and certain punishment. The experimental results did not support H5.

According to the uncertainty–identification theory, which mainly puts emphasis on the context-induced feelings of uncertainty (Hogg, 2007), people tend to strengthen their group identity and elicit more cooperative behavior to reduce self-uncertainty (Hohman & Hogg, 2015). Hohman and Hogg (2011) employed two experiments to investigate the role that uncertainty plays in reactions to mortality salience, and they found self-uncertainty plays an important role in ideological conviction and group behavior, which support the uncertainty–identity theory. Researchers also found that uncertainty has the power to strengthen identification with radical groups. Their results suggest that the effect of uncertainty was directly reflected on people’s intentions to engage in specific group behaviors. And group identification mediated participants’ behavioral intentions (Hogg et al., 2010b). In addition, Jung et al. (2016) employed South Koreans’ nested identity context to explore how people respond to uncertainty about a specific identity. They proposed that subgroup national identity strengthened superordinate identification. Based on the uncertainty–identification theory, what should be shown in this study is that, when participants received the uncertain punishment, their sense of uncertainty will increase, and they will be more inclined to cooperate; in addition, the proportion of a higher return amount will increase.

However, H5 was not verified. There is no significant difference in the cooperative behavior between uncertain and certain punishment. The reason for this result may be the effect of emotion on cooperative behavior. Psychologists have long proven that humans’ decision making depends on their current emotions (Bless et al., 1996; Schwarz & Clore, 1996/2006). Emotions are regarded to play an important role in the emergence and maintenance of cooperation. Joffily et al. (2014) have investigated the role of emotions in cooperation behavior. They found that a lower arousal and more positive valence are associated with participants’ greater contributions, whereas, triggered by greater arousal, one tends to make lower contributions. Moreover, Drouvelis and Grosskopf (2016) explored the effects of induced positive and negative emotions on cooperation behavior in a one-shot game. Their results also showed that negative emotions such as anger have a negative influence on shaping behavior in voluntary contribution. Participants contributed less in the “negative emotions” treatments (Drouvelis & Grosskopf, 2016). From the above findings, it was revealed that the reduction in contributions was a result of the induced emotions. Therefore, greater negative emotions were noted as the impact of contribution induction in this study. Influenced by the uncertain–identification theory, the contribution (i.e., the amount returned to investors in this study) should have been greater after uncertain punishment than after certain punishment. Since the results indicate that both the P2 index and the anxiety level were larger after uncertain punishment than certain punishment, this suggests that uncertain punishment arouses more negative emotions in participants. The greater negative emotion caused a negative impact on participants’ cooperative behavior, and, thus, leads to the reduction of their contributions. To some extent, the contribution increment caused by the strengthened identification and the contribution reduction led by the greater negative emotion may offset each other, which leads to the insignificance of participants’ cooperative behaviors in these two punishment conditions.

There is another reason why H5 was not supported. Because the index of behavioral change came from the following trial, where the participant had known whether the actual electric shock of the preceding trial was implemented or not. Then, the uncertainty was eliminated. The uncertain cue of the preceding trial could be regarded as certain shock or no-shock by participants, when they made the decision on payback.

**Figure 5:**
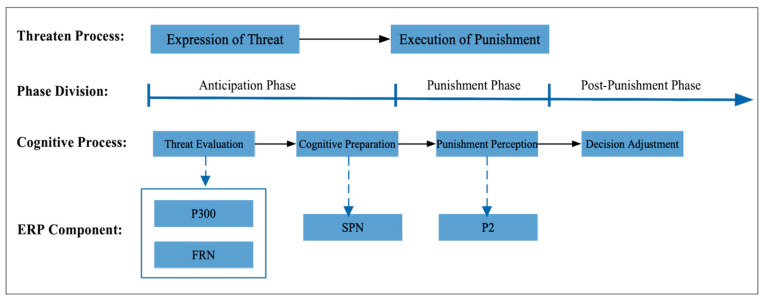
Threat punishment cognitive processing model.

Based on the findings of this study, we propose a comprehensive cognitive neural processing model for the quantitative measurement of punishment perception (see Figure 5).

The model delineates individuals’ perception and impact of punishment into four distinct stages:

(1) Threat Evaluation Stage: This initial stage involves the assessment of perceived threats. It is closely related to the individual’s attention mechanisms and the comparison between prior knowledge and the current threat. This stage can be effectively represented by the P300 component, which is indicative of attentional processes, and the feedback-related negativity (FRN), which reflects the neural response to feedback, particularly negative feedback. P300 and FRN serve as critical markers for understanding how individuals evaluate and react to potential threats.
(2) Cognitive Preparation and Expectation Stage: At this stage, the individual engages in cognitive preparation and forms expectations about the impending punishment. This stage can be measured using the stimulus-preceding negativity (SPN), which captures the anticipatory neural activity preceding the expected stimulus. SPN provides insights into the preparatory cognitive processes that occur as individuals brace themselves for potential adverse outcomes.
(3) Punishment Perception Stage: Upon receiving the punishment, the individual undergoes a stage of perceptual processing. This stage is characterized by the P2 wave, which is associated with cognitive matching processes and reflects the brain’s response to the actual experience of punishment. The P2 wave serves as an important index for quantifying the immediate perceptual impact of the punishment.
(4) Decision-Making Adjustment Stage: The final stage involves changes in decision-making and behavior in response to the punishment. These changes can be directly measured through behavioral indicators, providing a clear link between cognitive processes and observable actions. This stage is crucial for understanding how punishment influences subsequent behavior and decision-making.

By quantifying the cognitive processing changes at each stage of the threat of punishment, this model offers a robust framework for measuring and understanding punishment perception. The theoretical contributions of this study extend the existing literature on punishment by providing a detailed cognitive–neural mechanism underlying the perception and processing of punishment. From a practical standpoint, the findings offer valuable indicators for managers and policymakers to design and evaluate reward and punishment systems more effectively.

This model bridges the gap between individual psychological states, cognitive processing changes, and behavioral outcomes, providing a holistic view of the punishment process. It underscores the importance of a multi-faceted approach to studying punishment, integrating insights from cognitive neuroscience with practical applications in management and policy. In real life, punitive actions often involve significant costs and ongoing expenses. For instance, engaging in punitive competition with other companies or imposing economic sanctions on other countries requires investment in money, personnel, and resources. The greater the intensity of the punishment is, the more resources are consumed. Moreover, punitive measures can lead to “self-damage”, where the punisher also incurs some loss. Additionally, there may be retaliatory measures from the punished party, which can further increase the costs.

Thus, effective punishment in real-world scenarios requires careful cost consideration. By using uncertain punitive methods, such as varying the intensity, timing, or occurrence of the punishment, one can minimize costs while maintaining the effectiveness of the threat. For example, if a low-intensity punishment is preferred after cost evaluation, one can threaten with an “uncertain intensity” to achieve a threatening effect comparable to or greater than that of high-intensity punishment. If the other party does not heed the warning, only a low-cost, low-intensity punishment is needed. By strategically manipulating the uncertainty of the punishment, one can achieve a high-threat effect at a lower cost.

In this study, the probability of punishment delivery in the uncertain condition was fixed at 50%. Without manipulating this probability, it is not possible to thoroughly investigate how the degree of punishment uncertainty relates to brain activity, emotional responses, and behavior. Future studies should incorporate the systematic manipulation of uncertainty levels within the experiment, in order to more thoroughly investigate the relationship between uncertainty and punishment effects.

## 5. Conclusions

Concluding from the above, this study shows the differences between a certain and uncertain threat from the microscopic view. There were significant differences in neurophysiological indicators, but the differences in behavioral outcomes were not significant due to the great influence of emotion. Therefore, more moderate physiological punishments may be considered for future research and practical application. Uncertain punishment can realize both cost savings and an effective penalty at the same time. Uncertainty brings more negative emotion, and more pain.

## Figures and Tables

**Table 1:** Self-ratings of anxiety under different cues and electric shock.

Cue	Mean	Standard Error	95% Confidence Interval	
			Lower	Upper
NoShock	24.36	3.88	16.27	32.45
Shock	60.17	3.69	52.47	67.87
Uncertain-NoShock	73.16	3.9	65.02	81.30
Uncertain-Shock	69.6	3.35	62.61	76.59

**Table 2:** Results of repeated-measures ANOVA.

	Source	Sum of Squares	df	Mean Square	*F*	*p*	η^2^	Observed Power
Anxiety	Cue	31,406.81	1.54	20,422.13	44.91	0.000	0.692	1
	error(Cue)	13,985.53	30.76	454.70				
P300	Cue	487.29	1.34	363.67	11.81	0.001	0.371	0.956
	error(Cue)	825.14	26.80	30.79				
	Electrode	21.29	1.55	13.77	0.84	0.414	0.04	0.166
	error(Electrode)	506.15	30.94	16.36				
	Cue × Electrode	15.64	4.00	3.91	2.22	0.074	0.1	0.628
	error(Cue × Electrode)	140.77	80.00	1.76				
FRN	Cue	48.02	1.00	48.02	3.23	0.087	0.139	0.402
	error(Cue)	297.24	20.00	14.86				
	Electrode	58.27	1.82	31.94	7.93	0.002	0.284	0.922
	error(Electrode)	147.01	36.48	4.03				
	Cue × Electrode	12.30	3.00	4.10	3.90	0.013	0.163	0.802
	error(Cue × Electrode)	63.10	60.00	1.05				
SPN	Cue	1929.62	2.00	964.81	13.60	0.000	0.405	0.997
	error(Cue)	2838.34	40.00	70.96				
	Electrode	25.51	2.40	10.64	1.37	0.265	0.064	0.305
	error(Electrode)	373.11	47.94	7.78				
	Cue × Electrode	122.86	3.63	33.83	3.61	0.012	0.153	0.827
	error(Cue × Electrode)	680.95	72.64	9.38				
Pain-P2	Cue	4583.86	1.00	4583.86	59.69	0.000	0.749	1
	error(Cue)	1535.90	20.00	76.80				
	Electrode	1566.15	1.93	811.46	15.96	0.000	0.444	0.999
	error(Electrode)	1963.20	38.60	50.86				
	Cue × Electrode	10.21	2.07	4.94	0.82	0.453	0.039	0.182
	error(Cue × Electrode)	250.12	41.33	6.05				

Greenhouse–Geisser correction is applied if the assumption of homogeneity of variance is violated.

**Table 3:** Results of mixed-effects model on single-trial analysis.

Variable	Estimate	SE	*t*	df	*p*	95% CI	
						Lower	Upper
(Intercept)	26.875	2.489	10.799	1684	0.000	21.994	31.756
Amount	−0.312	0.119	−2.624	1684	0.009	−0.544	−0.079
isShocked	−2.887	1.875	−1.540	1684	0.124	−6.564	0.789
isCertainShock	37.776	2.273	16.619	1684	0.000	33.317	42.234
isUncertain	48.268	1.578	30.588	1684	0.000	45.173	51.363
300–600 ms Theta	0.037	0.039	0.930	1684	0.352	−0.041	0.114
300–600 ms Alpha	−0.065	0.036	−1.812	1684	0.070	−0.135	0.005
300–600 ms Beta	0.218	0.105	2.079	1684	0.038	0.012	0.423
800–1000 ms Theta	0.040	0.041	0.960	1684	0.337	−0.041	0.121
800–1000 ms Alpha	−0.044	0.040	−1.110	1684	0.267	−0.123	0.034
800–1000 ms Beta	0.085	0.089	0.955	1684	0.340	−0.090	0.260
1100–1300 ms Theta	−0.023	0.031	−0.732	1684	0.464	−0.083	0.038
1100–1300 ms Alpha	0.080	0.034	2.327	1684	0.020	0.013	0.147
1100–1300 ms Beta	0.030	0.049	0.624	1684	0.533	−0.065	0.126

## Data Availability

The datasets generated and analyzed during the current study are not publicly available due to privacy protection for the participants but are available from the corresponding author upon reasonable request.
